# The Promise of Patient Portals for Individuals Living With Chronic Illness: Qualitative Study Identifying Pathways of Patient Engagement

**DOI:** 10.2196/17744

**Published:** 2020-07-17

**Authors:** Maureen T Stewart, Timothy P Hogan, Jeff Nicklas, Stephanie A Robinson, Carolyn M Purington, Christopher J Miller, Varsha G Vimalananda, Samantha L Connolly, Hill L Wolfe, Kim M Nazi, Dane Netherton, Stephanie L Shimada

**Affiliations:** 1 Institute for Behavioral Health The Heller School for Social Policy and Management Brandeis University Waltham, MA United States; 2 Center for Healthcare Organization and Implementation Research Edith Nourse Rogers Memorial Veterans Hospital Bedford, MA United States; 3 Department of Population and Data Sciences University of Texas Southwestern Medical Center Dallas, TX United States; 4 Department of Medicine Boston University School of Medicine Boston, MA United States; 5 Center for Healthcare Organization and Implementation Research Boston VA Healthcare System Boston, MA United States; 6 Department of Psychiatry Harvard Medical School Boston, MA United States; 7 Department of Health Law, Policy, and Management Boston University School of Public Health Boston, MA United States; 8 Independent Consultant Coxsackie, NY United States; 9 Division of Biostatistics and Health Services Research Department of Population and Quantitative Health Sciences University of Massachusetts Medical School Worcester, MA United States; 10 Division of Health Informatics and Implementation Science Department of Population and Quantitative Health Sciences University of Massachusetts Medical School Worcester, MA United States

**Keywords:** patient engagement, patient portal, chronic disease, diabetes, secure messaging, veterans

## Abstract

**Background:**

Patients play a critical role in managing their health, especially in the context of chronic conditions like diabetes. Electronic patient portals have been identified as a potential means to improve patient engagement; that is, patients’ involvement in their care. However, little is known about the pathways through which portals may help patients engage in their care.

**Objective:**

Our objective is to understand how an electronic patient portal facilitates patient engagement among individuals with diabetes.

**Methods:**

This qualitative study employed semistructured telephone interviews of 40 patients living with diabetes since at least 2011, who had experienced uncontrolled diabetes, and had used secure messaging through a portal at least 4 times over 18 months. The interviews were recorded, transcribed, coded, and analyzed using primarily an inductive approach to identify how patients living with diabetes use an online health portal to support diabetes self-management.

**Results:**

Overall, patients who used the portal reported feeling engaged in their health care. We identified four pathways by which the portal facilitates patient engagement and some challenges. The portal provides a platform that patients use to (1) better understand their health by asking questions about new symptoms, notes, or labs, (2) prepare for medical appointments by reviewing labs and notes, (3) coordinate care between VA (Veterans Affairs) and non-VA health care teams, and (4) reach out to providers to request help between visits. Several patients reported that the portal helped improve the patient-provider relationship; however, aspects of the portal design may hinder engagement for others. Patients reported challenges with both secure messaging and access to medical records that had negative impacts on their engagement. Benefits for patient engagement were described by many types of portal users with varying degrees of diabetes control.

**Conclusions:**

Patient portals support engagement by facilitating patient access to their health information and by facilitating patient-provider communication. Portals can help a wide range of users engage with their care.

## Introduction

Patients play a crucial role in managing their health, and that role is even more central in the context of chronic conditions that require ongoing coping skills and self-management efforts [1,2]. Broadly, the term “patient engagement” refers to patients taking part in or actively participating in their care [3], including how patients work with health care providers and systems to manage and improve their health. Hibbard [4] conceptualized patient engagement as degrees of active involvement in their care, termed patient activation. Patient engagement can operate at multiple levels and exists on a continuum [5]. We focus primarily on the level of direct patient care where engagement can range from patients receiving information about a diagnosis to patients participating in treatment plan decisions [5]. Although it has been characterized in a variety of ways, a common thread is that patient engagement is a multi-faceted concept incorporating actions on multiple levels to promote patient-centered care and self-management.

Patient-facing eHealth technologies are often described as a means to improve patient engagement [6,7]. Online patient portals have received particular attention since their features often support healthcare system transactions (eg, prescription refills, scheduling), healthcare team communication, access to patient medical records, and the delivery of health education resources [7,8]. Previous studies of patients living with diabetes, for example, suggest that individuals who use online patient portals are more likely to achieve desirable outcomes, including improved glycemic control [9-12].

Few studies have attempted to specify how engagement may be realized through patient portals or the pathways through which portals may help patients engage in their care and self-management. One study of direct-release of test results via patient portals found that this access to information improved engagement by helping patients to monitor results over time and prepare before communicating with their provider [13]. However, a greater understanding of pathways through which a range of portal features support engagement is needed.

A framework for patient engagement by Barello and colleagues may help us understand the different ways in which a patient portal may support patient engagement [14]. This framework incorporates patients’ actions, thoughts, and feelings and considers patient engagement within three dimensions: emotional, cognitive, and behavioral [14]. In this model, the emotional dimension of engagement encompasses the patients’ emotional state and feelings about their care. The cognitive dimension captures patients’ understanding of their disease and treatment. The behavioral dimension of engagement captures patients’ actions to address their health.

In this paper, we examine whether and how an online patient portal supports patient engagement for individuals living with diabetes. We take a qualitative approach to this by reviewing patient portal experiences among veterans living with diabetes and describe several ways in which an electronic patient portal facilitates patient engagement.

## Methods

Data were collected in a more extensive study examining the role of patient portals in diabetes management, described previously [15]. Briefly, from November 2017 to January 2018, 1200 patients living with diabetes were invited to participate in a mailed survey about their diabetes self-management and use of the United States Department of Veterans Affairs (VA) online patient portal, My Health***e***Vet [16]. The My Health***e***Vet portal offers several features to help patients manage their care. Patients can request medication refills, schedule appointments, receive appointment reminders, communicate electronically with providers through secure messaging, and use the Blue Button feature to access portions of their medical records including labs and clinical notes [17].

All 1200 patients had a diabetes diagnosis in their record since at least 2011, had experienced uncontrolled diabetes in 2012 (mean HbA_1c_ >8.0% and less than 25% of the year with an HbA_1c_ <8.0%) and were actively engaged patient portal users who used secure messaging at least four times between January 2016 and June 2017. Since 2012, half had achieved glycemic control, defined as having mean HbA_1c_ <8% in 2016 and HbA_1c_ <8% for at least 75% of 2016, and half had not. We stratified each group based on urban/rural residence, according to the Rural-Urban Commuting Areas [18], and the presence of comorbid mental health diagnoses. Individuals were considered to have a comorbid mental health diagnosis if they had two outpatient diagnoses or one inpatient diagnosis within each diagnostic group. The diagnostic groups selected using International Classification of Diseases (ICD), 9th and 10th Revision (ICD-9 and ICD-10) codes were anxiety, bipolar, depression, personality disorder, post-traumatic stress disorder, schizophrenia, and substance use disorders. We received 449 completed surveys, of which 350 patients responded that they would be interested in participating in an interview about how they managed their diabetes. This study was approved by the Edith Nourse Rogers Memorial Veterans Hospital Institutional Review Board.

Purposeful sampling was used to identify and select patient portal users for telephone interviews. In total, 160 respondents provided positive responses to an open-ended survey question, “Can you tell us about an ‘A-Ha!’ moment when you realized you could use the My Health***e***Vet portal to better manage your diabetes?” We selected interviewees to represent a variety of responses to this and other survey items about My Health***e***Vet use. Interviewees were selected to represent those who used a variety of My Health***e***Vet portal features, those with controlled and uncontrolled diabetes, urban and rural patients, and those with and without comorbid mental health diagnoses. Women and minority veterans were oversampled to broaden the representation of patient demographics.

Forty telephone interviews were completed between February and May of 2018. Each interview was conducted by two researchers and averaged 70 minutes long. The interview covered how the patient managed their diabetes, including their feelings about the efficacy of their self-management, how they gathered and used information, how portal features and other technologies supported their diabetes management, and their suggestions for improving the portal. Interviewers were intentionally blinded to the patient’s glucose control at the time of the interview.

### Participants

The demographic and health characteristics of our patient sample, obtained from the survey data and health records, are reported in Table 1. The mean age of participants was 65.9 years. The majority were white (85%) and male (80%); 60% had a mental health diagnosis. Participants’ mean HbA_1c_ tested closest to the interview (recent HbA_1c_) was 8.2% (SD 1.4%), over our threshold for diabetes control (<8.0).

### Approach

All interviews were recorded, transcribed, and double-coded for multiple themes, using a coding scheme developed by the team. Several coders collaborated to establish the list of codes using both deductive and inductive thematic coding [23]. Deductive coding was used initially to create a list of preliminary codes from the topic areas of our interview guide. These codes included the benefits and challenges of each specific portal feature. Inductive codes were developed as coders reviewed the narrative, and new themes emerged from the interview transcripts. Coders met regularly to discuss the codes and themes, and to ensure consensus on meaning was achieved. Coding discrepancies were resolved with discussion among three authors (MTS, SLS, TPH). Coders were blinded to the interviewee’s glucose control status.

We selected eight codes related to portal use and patient engagement for in-depth analysis and reporting here. The following inductively developed codes were used to identify pathways for engagement: (1) patient-team relationship (portal use impact on the patient-healthcare team relationship); (2) empowerment (patients feeling empowered through portal use); (3) care collaboration (patients using the portal to coordinate care with their healthcare teams); (4) impact on care plan (how portal use changes patients’ care plans between visits); (5) clarification (patient-initiated communication through the portal for explanations of information). The following deductively developed codes were used to identify challenges to patient engagement: (1) secure messaging challenges, (2) medication refill challenges, (3) BlueButton challenges. The interview text excerpts identified by these codes were analyzed, and themes around how the patient portal facilitates engagement were elicited and used to organize our results.

**Table 1:** Sample characteristics (N=40).

Characteristic		Value
Diabetes Self-Management Questionnaire (DSMQ) Score^a^, mean (SD)		8.0 (0.79)
Mean diabetes self-efficacy score (DSES)^b^, mean (SD)		7.4 (1.6)
Recent HbA_1c_^c^, mean (SD)		8.2 (1.4)
Nosos risk score^d^, mean (SD)		2.4 (2.7)
Age (years), mean (SD)		65.9 (6.5)
Male, n (%)		32 (80)
**Race/Ethnicity**, n (%)		
	White	33 (85)
	Black	5 (13)
	Latino, n (%)	2 (5)
Rural, n (%)		21 (53)
**Health information**, n (%)		
	Has a mental health diagnosis	24 (60)
	Recent HbA_1c_ in control (<8.0%)	22 (55)
**Health literacy**^e^, n (%)		
	Inadequate	2 (5)
	Marginal	4 (10)
	Adequate	34 (85)
**Income ($US)**, n (%)		
	<$25,000	9 (23)
	$25,000-$49,000	10 (25)
	$50,000-$149,000	15 (38)
	>$150,000	3 (8)
	Income not reported	3 (8)

^a^Diabetes Self-Management Questionnaire (DSMQ): a global measure of diabetes self-management comprised of 16 items to assess activities related to glycemic control in patients with diabetes. Scaled scores range from 0-10 and higher values indicate more effective self-management [19].

^b^Diabetes Self-efficacy Scale (DSES): measures how confident patients are in their ability to do certain activities related to managing their diabetes. Scores range from 1-10 and higher values indicate higher self-efficacy [20].

^c^HbA_1c_: glycated hemoglobin.

^d^Nosos risk score: VA’s modified version of Medicare’s Hierarchical Condition Category. A measure of expected health care costs based on demographic, pharmacy, psychiatric and health care utilization data; mean for a population equals 1.0 and scores >1.0 indicate the patient is expected to have health care costs that much higher than the average VA patient [21].

^e^Health literacy: determined by the patient’s response to the question, “How often do you have someone help you read hospital materials? [22]” Health literacy was considered inadequate when patients responded “Often” or “Always”; “Sometimes” was considered marginal health literacy; “Never” was considered adequate health literacy.

## Results

Across all 40 patient interviews, 30 touched on concepts related to patient engagement through portal use. Overall, patients reported feeling engaged by their use of the portal. We describe this below, followed by illustrations of the four pathways of interaction with the portal that support patients’ feelings of engagement. Finally, we share patient insight on how portal functionality may hinder engagement. To further contextualize our data, we describe select patient characteristics after each illustrative quote.

### Feeling Engaged by Use of the Portal

Patients explained that the portal helped to support their engagement with their care and improve their health by having a provider available to give consistent feedback. One patient reported that interacting with providers through the portal improved their attitude and health:

The best thing I ever did was when I enrolled in (My HealtheVet). It helped me be in better control of my attitude, my depression, my diabetes. When I deviate a little bit left or right, there’s always somebody on the other end going, ‘uh oh, you need to go down that straight and narrow path again.’ It’s a great program. (60-year-old Latino male, white, urban, mental health diagnosis, recent HbA_1c_ 7.3, in control)

Patients reported feeling that interacting with providers through the portal helped build patient-provider relationships and that these relationships were vital to receiving better care:

The more interest you show, the more interest they show in you. I think the secure messaging helps you establish that kind of relationship. Like, ‘Oh here’s a guy that’s trying to take care of himself, let’s help him. (82-year-old white male, rural, no mental health diagnosis, recent HbA_1c_ 7.5, in control)

### How the Portal Supported Patient Feelings of Engagement

Our analysis identified four key pathways by which the portal supported patients’ engagement in their care and some difficulties with the portal that may hinder engagement. The portal provides support for patients to (1) work to better understand their health, (2) prepare for medical appointments, (3) coordinate care and share health information between VA and non-VA health care teams, and (4) reach out to providers to request help between visits. These pathways are illustrated below.

#### Working to Better Understand Their Health

Reading clinical notes and test results helps patients understand their health information in their own time, gauge the seriousness of health issues they have, keep things in perspective, and make decisions about appropriate next steps. For example, patients reported that reviewing clinical notes using the Blue Button portal feature after an appointment helped them to understand what their provider was telling them. “It doesn’t always sink in right away what they are telling you. So I’ve used the Blue Button notes.” (69-year-old white male, rural, no mental health diagnosis, recent HbA_1c_ 7.5, in control)

Making test results available through the patient portal provides an opportunity for patients to be proactive based on test results:

I think that was the “A-ha” moment when I said, ‘now I can see my results of 50 different tests’. And all at once and go over them and pick out the ones that are too high or too low. Then if I see something way off, I can make an appointment. (72-year-old black male, urban, no mental health diagnosis, recent HbA_1c_ 6.9, in control)

Sharing health information through patient portals can also cause some distress when patients do not understand the information. One participant described a situation like this, but said they use the secure messaging feature to obtain more information and reassurance from their provider:

I’ve had issues where I [view a test result] and it sounds really bad but the note says it’s no big deal so I would send a secure message to my doctor and ask them why they’re not concerned about this level being high, and she would explain it more to me. So that’s helped. (51-year-old black female, urban, mental health diagnosis, recent HbA_1c_ 9.2, not in control)

Patients reported finding the portal helpful because they could ask questions before they forget. The portal helps reduce the chance of a patient forgetting a health concern or inquiry by enabling them to send providers questions via secure messaging right when patients think of them:

Sometimes you can’t get an appointment for a week or so and by then you’ve forgotten what question you have! So it’s so much easier just writing it down in that secure message and sending it off before you forget what you want to ask. (61-year-old white female, urban, no mental health diagnosis, recent HbA_1c_ 7.0, in control)

Patients also used the information available through the portal together with information available from other sources to better understand their conditions:

There’ve been times when I look at my blood work and I see something I don’t understand; medical jargon. I’ve been able to Google it and find out what it means. That’s empowering. (51-year-old black female, urban, mental health diagnosis, recent HbA_1c_ 9.2, not in control)

#### Preparing for Medical Appointments

Patients described reviewing notes to prepare for their visit to be proactive about their care. One patient reported using the patient portal to prepare for their appointment so that they would be able to ask thoughtful and helpful questions:

I’m always checking on my lab results. I ask the doctor if something’s high or low because I know the results before I go in and see her. That’s what I like about [My HealtheVet], I know the lab results, I know what questions to ask. (72-year-old black male, urban, no mental health diagnosis, recent HbA_1c_ 6.9, in control)

Another patient described using the portal to review information and vocabulary to engage more meaningfully during appointments in the conversations with their provider:

It’s easier to sit there and look at [My HealtheVet] and have an idea of what’s going on before you talk to the people who know more about it than you do… So that I have an understanding of what terms that they’re using. So that they aren’t snowballing me or going over my head. (69-year-old white male, rural, no mental health diagnosis, recent HbA_1c_ 7.5, in control)

#### Coordinating Care and Sharing Health Information Between VA and Non-VA Health Providers

Having access to the patient portal puts control over health information into the hands of patients. When patients want to share information between providers, patients can send information themselves. Patients reported sharing lab results between VA and non-VA providers to avoid duplicate labs and unnecessary testing. This access saves time and health care resources.

With the VA I usually get labs done every 2 months, give or take. Privately it’s probably about the same, every 2-3 months. It goes back and forth. That’s the nice thing about this. Go from private to the VA. VA to private. You can take that information and if the doctors need it or want it, you can transfer it to them. (71-year-old white male, rural, mental health diagnosis, recent HbA_1c_ 8.2, not in control)

Patients also reported using the portal to coordinate care between VA and non-VA providers because the providers do not coordinate themselves. When patients have to take on the care coordination role, having information in writing from each doctor supports accurate sharing of information between providers:

I have a VA doctor and I have one through Medicare, I’ve got to be very careful that I have both of them in agreement. So what I end up doing is if one of them recommends a different type of medication … I can email both of them … they don’t seem to want to talk, for whatever reason they don’t talk on the phone. So I will literally copy and send emails back and forth with the pros and cons … I have something in writing that I can actually communicate back, rather than me trying to remember. (68-year-old white male, urban no mental health diagnosis, recent HbA_1c_ 7.8, in control)

#### Reaching Out to Providers to Request Help or Changes to Care Plan Between Visits

Close collaboration with providers may facilitate improved health and reduce the amount of in-person medical visits, which may be especially burdensome for rural patients. Patients reported using the portal to reach out to providers for information about how to address symptoms and manage their blood sugar levels. Patients described they found secure messaging supportive as a tool to reach out to their providers to understand why their blood sugar is too low or too high and facilitates their ability to receive guidance about what to do differently. Patients use secure messaging to help them as they work to manage their health and understand why they may have certain symptoms,

I sent a question to my care team [through secure messaging] asking about what I’m doing wrong because I’m ending up with morning [blood sugar] numbers that are too low…I like that because they’re usually pretty prompt at getting back to me. (59-year-old white male, rural, no mental health diagnosis, recent HbA_1c_ 6.5, in control)

Variations of this comment were heard from many patients:

If I thought my [A_1c_] was out of whack or … that I think it’s high, then I might say something. Especially when I don’t understand why it’s high. (72-year-old black male, urban, no mental health diagnosis, recent HbA_1c_, 6.9, in control)

Between visit communication between patients and providers through secure messaging affords an opportunity to more quickly address issues, including potentially changing medication management plans, to try to gain control over blood sugar:

I could send the readings via secure message and, then they’d say, ‘Okay, well you can drop this dose or you need to add this dose. (60-year-old white female, rural, mental health diagnosis, recent HbA_1c_ 9.9, not in control).

Patients reported that they used portal interactions to advocate for themselves and be proactive about getting what they need, including asking about how to manage blood sugar better:

If I notice that my blood sugar stays high and I can’t seem to regulate it, I use My HealtheVet to do the secure messaging and I’ll send my care provider [a message]. I usually let it go for a couple of days so that I can see an average. And, then I’ll contact them and maybe they’ll contact me right back and say, ‘Listen, I need you to increase it by two units. And let’s see if we got you on track now.’ (61-year-old black male, urban, mental health diagnosis, recent HbA_1c_ 7.7, in control)

Some patients also reported these interactions reduced the number of in-person visits required.

If I punched in high numbers into telehealth, [my doctor] would message me, find out what I was doing and tell me what I needed to adjust. I have to say that a lot of [my motivation to use My HealtheVet] had to do with being able to have more interaction directly with my doctor straight from my house. I almost don’t even have to go to the VA anymore. (54-year-old white male, rural, mental health diagnosis, recent HbA_1c_, 7.2, in control)

Patients also found secure messaging to be an efficient way to ask about how to address medication side effects they were experiencing:

You know, I can ask her, ‘This new medication is making me a little light-headed or whatever. What should I do.’ And I get an answer the next day. (56-year-old white male, rural, mental health diagnosis, recent HbA_1c_ 9.7, not in control)

### Portal Functionality Hinders Engagement

Patients identified some issues with portal features that may have dampened their engagement. First, multiple patients reported being unable to use secure messaging with all of their providers. Patients do not always understand that this is a portal design issue; some misinterpret this as a purposeful act on the part of their providers and may see it as a form of rejection:

On secure messaging I have 6 groups of different people including my primary doctor and my pharmacist. I used to have my hepatologist and somehow they took that off of there. I do a lot of work through my hepatologist and I can’t secure message him because he’s not on my board anymore, which makes me very mad. (67 year old male, urban, mental health diagnosis, recent HbA_1c_ 6.1, in control)

Second, patients reported that they could not send secure messages to individual providers, but instead had to message the team and that their uncertainty about who might read the message made them uncomfortable.

They call it secure messaging but it doesn’t go to the specific person, it just goes to the department and then it gets trickled down from there. So you don’t really know who’s going to be reading all that. I have to write to the purple team, not my primary doctor. (57-year-old American Indian/Alaska Native female, urban, no mental health diagnosis, recent HbA_1c_ 7.4, in control)

Finally, patients described challenges with the BlueButton feature that may hinder engagement, either by preventing access to information or generating frustration so that patients stop trying to engage. One patient said the information in BlueButton is not helpful and feels impersonal, “There is a lot of it that feels cookie-cutter, nothing new” (54-year-old white male, rural, no mental health diagnosis, recent HbA_1c_ 7.1, in control). Several patients reported the interface is difficult to navigate, and they expressed frustration with not knowing how to download their notes. Such challenges prevent them from obtaining information that may facilitate engagement. One reported, “finding medical record notes, that’s been hard for me. I couldn’t find that so I just said the heck with it.” (72-year-old white male, urban, mental health diagnosis, recent HbA_1c_ 8.1, not in control)

## Discussion

Through 40 interviews with patients with diabetes who used a patient portal, we engaged in a wide-ranging discussion regarding patient engagement and pathways by which portals may facilitate engagement. Access to detailed health information in the portal facilitates engagement by allowing patients to learn about their condition, remember information from provider visits that they otherwise may forget, and prepare for medical appointments. Tools to facilitate communication through the portal allow patients to partner with their providers to manage their health. Many patients found secure messaging and Blue Button features supportive of engagement; difficulties that patients reported with these features may, by extension, be barriers to patient engagement.

Patient narratives in this study align with Barello and colleagues’ framework [14] of three engagement dimensions (emotional, cognitive, and behavioral) and highlight an interconnectedness between cognitive and behavioral engagement. In terms of emotional engagement, patients felt that interacting with providers via the portal improved the patient-provider relationship. Portal use cognitively engaged patients to understand their health by using portal tools such as secure messaging to ask questions and Blue Button to review clinical notes. Patient narratives revealed how cognitive engagement is translated into behavior. Access to information through the patient portal led patients to engage in behaviors to support their health and self-management. Actions facilitated by the portal’s information and communication platform included preparing for visits, coordinating care among providers, and making lifestyle or medication changes between visits.

There are concerns in the literature that eHealth initiatives may worsen disparities [24] or weaken patient-provider relationships [12]. Disparities could worsen if vulnerable groups have less access to technology that promotes engagement or if vulnerable populations are less likely to use patient portals [25]. Our data suggest varied backgrounds and degrees of glucose control among patients using the portal to enhance their engagement. Portal benefits for engagement seem to be experienced by all types of patients, including individuals in urban and rural settings, with and without mental health conditions, those with diabetes in control, and those with diabetes not in control. Furthermore, several patients who were not in control described activities related to engagement. A portal may offer further opportunities to work with these patients to improve diabetes control. However, engaging patients via portal use does not necessarily indicate they will have better outcomes. In this analysis, we do not measure whether engagement facilitated by the portal influenced diabetes outcomes or patient satisfaction scores or whether this varied based on additional patient characteristics. The interviews were overwhelmingly positive about the benefits of the portal and suggested patient satisfaction is improved among those who use it. Future research should explore whether portal engagement predicts health outcomes and patient satisfaction and whether the findings are consistent among patients with other chronic conditions.

Patient-provider relationships are essential for individuals with chronic conditions like diabetes. There is some concern that portals may hurt the patient-provider relationship if portal interactions replace face-to-face interactions [12,26]. However, in our study, patients described the portal interactions between patient and provider as a key factor that facilitated their engagement. Some patients reported feeling that their interactions with providers through the portal help strengthen their relationship with providers. A point of caution: we must recognize the unintended consequence that a portal’s design may have on patient perceptions of their provider’s willingness to communicate with them. In our analysis, most patients found the portal helpful, but the design of the secure messaging feature led some patients to feel their providers may not want to communicate with them. Employing human-centered design may be one strategy to help mitigate portal design issues by engaging patients and providers early in the design process. One patient also complained about the impersonal, “cookie-cutter” nature of some clinical notes, which could make patients feel like their providers didn’t know them as individuals, or that the notes did not capture the essence of what was discussed during the clinical encounter. Future qualitative work should continue to explore patient and provider perspectives on how portals affect their relationships and could examine provider attitudes and approaches to the portal as a possible moderator of patient engagement.

This study has several limitations. The study population was limited to United Stated military veterans with diabetes who used the My Health***e***Vet patient portal. We found overwhelmingly positive comments regarding the portal, perhaps because all interviewees were portal users. Our study was not designed to determine the prevalence of patient engagement, but our findings do reveal potential ways in which a portal may facilitate engagement.

In this study, patient narratives helped identify pathways by which a portal may facilitate patient engagement. Patients found the portal helped strengthen their relationship with providers and helped the patient feel engaged. Patients reported finding the portal useful for receiving help in managing symptoms, coordinating their care, and learning about their health. Patient portal users included a variety of individuals who described the engagement benefits of the portal. The group included individuals living in urban and rural settings, those with and without mental health conditions, and those with controlled and uncontrolled diabetes. Thus, the portal may help a wide range of portal users engage with their care.

## Abbreviations

DMSQ: Diabetes Self-Management Questionnaire
DSES: Diabetes Self-efficacy Scale
ICD: International Classification of Diseases
VA: Veterans Affairs
